# Feasibility Trial of an eHealth Intervention for Health-Related Quality of Life: Implications for Managing Patients with Chronic Pain During the COVID-19 Pandemic

**DOI:** 10.3390/healthcare8040381

**Published:** 2020-10-01

**Authors:** John C. Licciardone, Vishruti Pandya

**Affiliations:** Department of Family Medicine, University of North Texas Health Science Center, Fort Worth, TX 76203, USA; vishruti.pandya@my.unthsc.edu

**Keywords:** COVID-19, eHealth, chronic low back pain, randomized controlled trial, health-related quality of life, low back pain intensity, back-related disability

## Abstract

Purpose: This study was conducted to determine the feasibility of providing an eHealth intervention for health-related quality of life (HRQOL) to facilitate patient self-management. Methods: A randomized controlled trial was conducted from 2019–2020 within the Pain Registry for Epidemiological, Clinical, and Interventional Studies and Innovation. Eligible patients included those with chronic low back pain and a SPADE (sleep disturbance, pain interference with activities, anxiety, depression, and low energy/fatigue) cluster score ≥ 55 based on the relevant scales from the Patient-Reported Outcomes Measurement Information System instrument with 29 items (PROMIS-29). Patients were randomized to the eHealth treatment group, which received a tailored HRQOL report and interpretation guide, or to a wait-list control group. The primary outcome was change in the SPADE cluster score, including its five component scales, over 3 months. Secondary outcomes were changes in low back pain intensity and back-related disability. Treatment effects were measured using the standardized mean difference (SMD) in change scores between groups. The eHealth intervention was also assessed by a survey of the experimental treatment group 1 month following randomization. Results: A total of 102 patients were randomized, including 52 in the eHealth treatment group and 50 in the wait-list control group, and 100 (98%) completed the trial. A majority of patients agreed that the HRQOL report was easy to understand (86%), provided new information (79%), and took actions to read or learn more about self-management approaches to improve their HRQOL (77%). Although the eHealth intervention met the criteria for a small treatment effect in improving the overall SPADE cluster score (SMD = 0.24; *p*= 0.23) and anxiety (SMD = 0.24; *p* = 0.23), and for a small-to-medium treatment effect in improving depression (SMD = 0.37; *p* = 0.06) and back-related disability (SMD = 0.36; *p* = 0.07), none of these results achieved statistical significance because of limited sample size. Conclusion: Given the feasibility of rapid online deployment, low cost, and low risk of adverse events, this eHealth intervention for HRQOL may be useful for patients with chronic pain during the COVID-19 pandemic.

## 1. Introduction

Many discretionary health care services were curtailed throughout the United States in March 2020 because of the national emergency attributable to the declaration by the World Health Organization of the pandemic of coronavirus disease (COVID-19) caused by the novel virus known as SARS-CoV-2 [1]. Consequently, the pandemic hastened the development and assessment of remotely supported medical services for a variety of non-urgent chronic conditions. Patients with chronic pain represent a large segment of the population at high risk of adverse health outcomes if they are unable to access appropriate medical management during the pandemic. Even before the pandemic, the Centers for Disease Control and Prevention reported that about 50 million adults in the United States suffer from chronic pain, including 20 million having high-impact chronic pain that interferes with work or life most days or every day [2].

A variety of remotely supported pain management approaches under the umbrella of telehealth have been considered for deployment during the COVID-19 pandemic, including eHealth, mHealth, virtual reality, augmented reality, remote treatment, and digital therapeutics [3]. The World Health Organization defines eHealth as the cost-effective and secure use of information and communications technologies in support of health and health-related fields, including health care services, health surveillance, health literature, and health education, knowledge and research [4]. Correspondingly, the Federal Pain Research Strategy has advocated research to study how technology may provide patients with health education that emphasizes self-help strategies to prevent, cope with, and reduce the impact of pain [5]. Therein, an important element of research involves the development of interventions aimed at persons having chronic pain conditions, with the goal of maintaining or improving health-related quality of life (HRQOL) and function [5,6]. The National Pain Strategy, which was guided by findings and recommendations from the report of the Institute of Medicine on Relieving Pain in America [7], states that patient self-management programs can improve HRQOL and are important components of chronic pain prevention and management [8].

The Patient-Reported Outcomes Measurement Information System (PROMIS) was developed with support from the National Institutes of Health for research on physical function, anxiety, depression, low energy/fatigue, sleep disturbance, participation in social roles and activities, and pain interference with activities [9]. Elements of the PROMIS-29 instrument for HRQOL involving physical function, depression, sleep disturbance, and pain interference with activities have been recommended by the National Institutes of Health Task Force as part of a minimum dataset to be used in conducting research on chronic low back pain [10]. The SPADE (sleep disturbance, pain interference with activities, anxiety, depression, low energy/fatigue) cluster derived from the PROMIS-29 has also been used as a measure of overall symptom burden in patients with chronic pain [11].

Health-related quality of life may be an attractive paradigm for guiding and improving chronic pain management because it is patient centered and may be more readily understood by patients than other more technical information, such as the results of laboratory tests or imaging studies. However, HRQOL measures have not been widely adopted in health care settings or by patients for self-management. The primary aim of this study was to determine the feasibility of providing a simple eHealth intervention pertaining to HRQOL to facilitate patient self-management.

## 2. Methods

### 2.1. Registry Overview and Study Design

The Pain Registry for Epidemiological, Clinical, and Interventional Studies and Innovation (PRECISION Pain Research Registry) was established in 2016 and subsequent operations were guided by principles recommended by the Agency for Healthcare Research and Quality [12]. A historical overview of the registry has been published [13]. We conducted a preliminary randomized controlled trial to study the feasibility of an eHealth intervention to improve the HRQOL of patients with chronic low back pain within the registry. The trial was approved by the North Texas Regional Institutional Review Board (protocol #2015-169) and registered with ClinicalTrials.gov (Identifier NCT04060953). In addition to determining the feasibility of providing the eHealth intervention for HRQOL to registry patients, the trial aimed to assess preliminary measures of the value and utility of the intervention and its short-term effect sizes. Patients for this feasibility trial were enrolled during the period from mid-August 2019 through mid-October 2019 and then followed for 3 months. All patients provided written informed consent prior to participating in the trial. They were given their usual registry compensation for providing trial baseline data (either $50 or $25, respectively, depending on whether these data were collected at the initial registry encounter or at a subsequent quarterly encounter) and outcomes data ($25). An additional incentive ($10) was provided for completing the survey of the value and utility of the eHealth intervention.

### 2.2. Inclusion and Exclusion Criteria

To be considered for inclusion in the trial, patients must have been 21 to 79 years of age at the time of registry enrollment and have had sufficient English language proficiency to complete the registry case report forms independently or with assistance from registry staff. Additionally, patients must have met the diagnostic criteria for chronic low back pain established by the National Institutes of Health Task Force [10] at the time of registry enrollment. These criteria require that patients report having low back pain for at least the past 3 to 6 months and with a pain frequency of at least half of the days during the past 6 months. Finally, patients must also have reported poor HRQOL immediately prior to enrolling in the trial. The latter was assessed using the SPADE cluster derived from the PROMIS-29 instrument [11]. Crude data for the five scales that comprise the SPADE cluster were transformed and standardized using scoring algorithms that were normed with a mean of 50 and SD of 10, based on the United States general population [9]. The SPADE cluster score was then computed as the mean of the five scale scores, with a mean score ≥ 55 required for inclusion in the trial. Patients with such scores are highly impacted by chronic pain and likely to have substantial deficits in multiple aspects of HRQOL that may be amendable to improvement. Patients were excluded from the trial if they reported being pregnant or institutionalized.

### 2.3. Experimental and Control Treatments

Patients were randomized to either the experimental or control treatment group using a random number generator. The experimental treatment group received the eHealth intervention consisting of a HRQOL report that was tailored to patients based on their scores for the SPADE cluster and each of its five component scales. The report included a graphic summary of scores for each measure and interpretation guide. A sample report is provided in the Supplementary Materials File S1. The control treatment group was placed on a waiting list to receive the eHealth intervention following the trial. Patients in both groups continued to receive their usual care for low back pain throughout the trial.

### 2.4. Survey of the Value and Utility of the eHealth Intervention

Patients in the experimental treatment group were surveyed 1 month following randomization to measure the value and utility of the eHealth intervention and patient behavior in response to it. The primary areas of interest included the value of the HRQOL report on a numerical rating scale (NRS) from 0 (not valuable at all) to 100 (extremely valuable); perceptions of the report (ease of understanding and acquisition of new information about HRQOL); and actions taken in response to the report (reading and learning about or implementing self-management approaches to improve HRQOL).

### 2.5. Outcome Measures

Outcomes were assessed 3 months following randomization. The primary outcome measure was the SPADE cluster score, including its five component scales. Secondary outcomes included low back pain intensity and back-related disability. Low back pain intensity was measured with an NRS for the average pain intensity over the past 7 days, ranging from 0 (no pain) to 10 (worst pain). Back-related disability was measured with the Roland-Morris Disability Questionnaire (RMDQ) [14], ranging from 0 to 24.

### 2.6. Sample Size

A total of 102 patients were included during the limited 2-month enrollment period. Detection of a small-to-medium effect size for the experimental treatment vs. control treatment, as manifested by a standardized mean difference (SMD) = 0.35 for change on the SPADE cluster score over the course of the trial, would have required 258 patients (129 patients in each treatment arm) to achieve 80% statistical power in intention-to-treat analysis [15]. Thus, the trial was not adequately powered to conduct definitive hypothesis tests pertaining to efficacy. However, this trial was performed primarily for planning purposes to determine the feasibility of providing the eHealth intervention, to assess preliminary measures of its value and utility, and to estimate its short-term effect sizes. These aspects of the trial have been used to plan and implement a larger follow-up trial, known as Optimizing Chronic Pain Management Through Patient Engagement with Quality-of-Life Measures (OPTIQUAL, ClinicalTrials.gov Identifier NCT04168437), which is projected to run through August 2021.

### 2.7. Statistical Analysis

The baseline characteristics of patients in the experimental and control treatment groups were measured using standard descriptive statistics and compared using contingency table methods for dichotomous variables and the Student’s t-test for continuous variables. Mean change scores for the primary and secondary outcome measures from baseline to the 3-month follow-up were used to compare the experimental and control treatment groups. Change scores were reversed so that a positive score represented an improvement in each measure (i.e., decreased HRQOL deficits, low back pain intensity, and back-related disability). Because the trial was not powered to detect treatment effects at or below the threshold for a small-to-medium effect size (SMD = 0.35), statistical significance was of secondary interest compared with the magnitude of the effect size of the eHealth intervention for each primary and secondary outcome measure [16]. As with change scores, positive values for effect sizes represented better outcomes over 3 months with the eHealth intervention as compared with being placed on the wait list. Effect sizes were classified using the following SMD criteria based on the estimated percentage of patients in the experimental treatment group expected to have superior outcomes in comparison with patients in the control treatment group: trivial, SMD < 0.20 (<58% superiority); small, 0.20 ≤ SMD ≤ 0.34 (61% superiority); small-to-medium, 0.35 ≤ SMD ≤ 0.49 (67% superiority); medium, 0.50 ≤ SMD ≤ 0.64 (72% superiority); medium-to-large, 0.65 ≤ SMD ≤ 0.79 (77% superiority); and large, SMD ≥ 0.80 (≥ 79% superiority) [16] Data were managed and analyzed with the IBM SPSS Statistics software package (Armonk, NY, USA, version 25). All hypotheses were assessed at the 0.05 level of statistical significance using 2-sided tests.

## 3. Results

**Figure 1:**
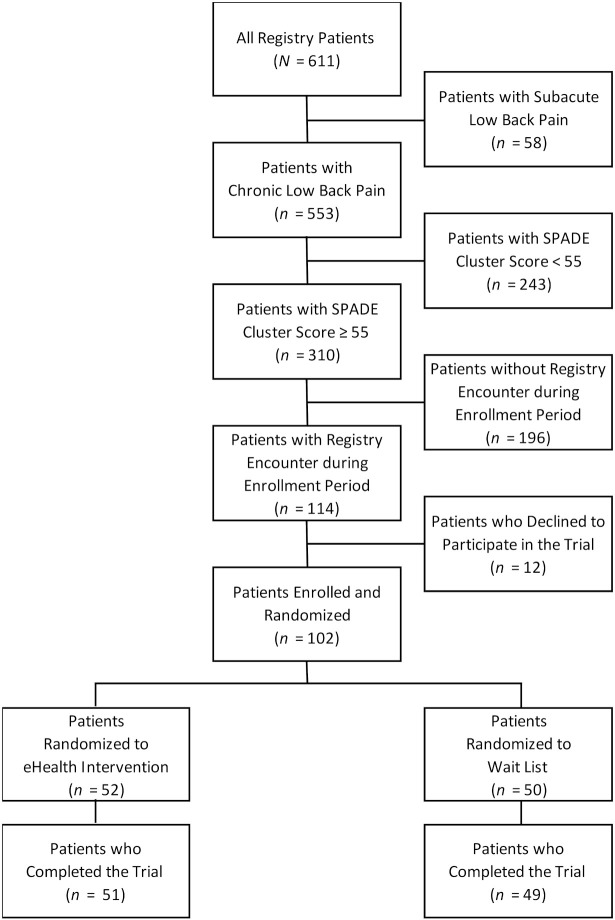
CONSORT diagram showing patient flow through the trial. SPADE denotes sleep disturbance, pain interference with activities, anxiety, depression, low energy/fatigue.

A total of 100 (98%) of the 102 randomized patients completed the trial (Figure 1). There were two (4%) patients who were unable to receive the eHealth intervention via online delivery and received it in person. The mean age of patients at baseline was 51.0 years (SD = 13.3 years), and 86 (84%) were women. The mean baseline SPADE cluster score was 61.6 (SD = 4.2). The mean scores for the NRS for low back pain intensity and RMDQ for back-related disability were 6.1 (SD = 1.8) and 16.8 (SD = 4.8), respectively. Randomization was successful in achieving balanced treatment groups, as there were no significant differences between patients in the experimental and control treatment groups in any of the 30 baseline characteristics except the SPADE depression scale score (Table 1).

The mean value rating of the eHealth intervention was 62.9 (SD = 29.0) (Table 2); however, the ratings were negatively skewed (median value = 74). A majority of patients who received the eHealth intervention agreed that the HRQOL report was easy to understand after reading the interpretation guide (86%). A majority also agreed that the report provided information about their HRQOL that they did not previously know (79%) and began reading or learning about self-management approaches to improve HRQOL (77%). Many also began new programs to improve their HRQOL (41%).

There were no significant differences between the experimental and control treatment groups in any primary or secondary outcome (Table 3). The changes in the SPADE cluster score with the eHealth intervention met the criterion for a small treatment effect (SMD = 0.24). Change scores for the eHealth intervention also met the criterion for a small-to-medium treatment effect for depression (SMD = 0.37) and back-related disability (SMD = 0.36). Correspondingly, these findings were associated with trends toward statistical significance favoring the eHealth intervention with regard to depression (mean change, 1.9; 95% CI, −0.1–3.9 vs. −1.1; 95% CI, −3.6–1.4; *p* = 0.06) and back-related disability (mean change, 0.9; 95% CI, −0.3–2.1 vs. −0.4; 95% CI, −1.2–0.4; *p* = 0.07). No adverse events were reported during the trial. Missing data were not imputed because 3-month follow-up was virtually complete.

## 4. Discussion

The eHealth intervention yielded several small or small-to-medium treatment effects over 3 months of follow-up in patients with chronic low back pain. Small treatment effects were observed for the SPADE cluster score for overall HRQOL and for anxiety. Small-to-medium treatment effects were also observed for the SPADE component scale of depression and for the secondary outcome of back-related disability. Interestingly, low back pain intensity did not trend in the direction of improvement with the eHealth intervention. These findings suggest that the eHealth intervention more specifically targeted HRQOL outcomes, and the related aspect of back-related functioning, rather than low back pain intensity. The importance of improving HRQOL in patients with pain has been emphasized in the Federal Pain Research Strategy [6]. Moreover, some have even questioned the appropriateness of using pain intensity to guide chronic pain management [17,18]. eHealth interventions aimed at improving HRQOL, such as that studied herein, may hold promise for chronic pain management during the COVID-19 pandemic.

Although the observed treatment effects for changes in overall HRQOL (including the component scales involving depression and anxiety) and back-related disability were considered to be of small or small-to-medium effect size, it is important to note that they were observed within only 3 months of randomization using a simple intervention that was tailored to individual patients and administered online at minimal cost and without reported adverse events. Most research on pharmacological therapy for chronic low back pain has focused on such outcomes as pain intensity, back-related disability, and adverse events. A systematic review and meta-analysis of nonsteroidal anti-inflammatory drugs, the recommended first-line pharmacological interventions for chronic low back pain [19,20] found treatment effects that were either slightly above or below the threshold for a small effect size (i.e., weighted mean difference = 10% of the measurement scale [21]) based on the reported findings for pain intensity and back-related disability outcomes over 3 months vs. placebo comparator [22]. However, that study also found a significantly greater risk of adverse events with nonsteroidal anti-inflammatory drugs [22].

Our PRECISION Pain Research Registry has shown decreased utilization of various recommended non-pharmacological treatments for chronic low back pain within the first three months of the COVID-19 pandemic in the United States [23]. Specific problems attributable to the COVID-19 pandemic often manifest themselves as HRQOL deficits, including those relating to physical functioning, deconditioning, pain interference with activities, anxiety, and depression [24]. Consensus recommendations from an international expert panel on caring for pain patients during the COVID-19 pandemic indicate that the biopsychosocial management of pain should consider online self-management programs that integrate components of a healthy lifestyle [25]. Thus, the eHealth intervention studied herein, which focuses on HRQOL, may be a viable complement or alternative for patients with chronic low back pain during the COVID-19 pandemic. Indeed, in the absence of suitable alternatives, telehealth modalities such as eHealth interventions have been recommended despite clear evidence of clinical benefit because these treatments carry very low risks [26].

As eHealth interventions for chronic pain are likely to remain after the COVID-19 pandemic ends [27], more research is needed on such interventions to improve HRQOL in patients with chronic low back pain. A clinical trial that was similar to ours in some aspects of study design, including the use of SPADE cluster score profiles to improve HRQOL in patients with chronic low back pain over 3 months, aimed the experimental treatment at clinicians rather than patients [28]. The investigators attributed the lack of significant improvement in SPADE cluster scores in their adequately powered trial to various factors that may have hampered primary care clinicians, including inadequate time, lack of resources (e.g., clinical team members), lack of extra patient contacts, and other competing demands. Additionally, they cited the need for simultaneous systems support to more effectively implement the intervention in clinical practice and to monitor the outcomes using electronic health records.

Our intervention was aimed at patients primarily to help them initiate self-management of chronic low back pain, particularly involving HRQOL. A majority of patients in our trial who received the eHealth intervention indicated that the report was easy to understand and provided new information about their HRQOL. Within only 1 month, a majority of patients also began reading and learning more about self-management approaches to improve their HRQOL. However, a shortcoming of the intervention was that standardized and validated information about improving HRQOL was not provided directly to patients. Thus, we could not determine if patients accessed self-management strategies that were appropriate for their HRQOL deficits and likely to result in improved outcomes at 3 months.

Our feasibility trial had several strengths. First, it involved a pragmatic approach using registry patients who received their usual care for chronic low back pain from community-based providers. Second, based on recommendations from the National Institutes of Health Task Force [10], diagnostic criteria for chronic low back pain and valid and reliable clinical outcome measures were used. Third, there was virtually complete follow-up at 3 months. Limitations of our trial included inadequate sample size and insufficient length of follow-up to explore long term effects of the eHealth intervention. In addition, two patients who were unable to access the online HRQOL report received it in person.

## 5. Conclusions

In summary, the present study demonstrated the feasibility of rapidly deploying an eHealth intervention for HRQOL in patients with chronic low back pain. A majority of patients who received the eHealth intervention indicated that the report was easy to understand, provided new information, and prompted them to read or learn more about self-management approaches to improve their HRQOL. The intervention yielded small or small-to-medium treatment effects over 3 months in several aspects of HRQOL. Given its relative ease, rapidity, and low cost of deployment, its low risk of adverse events, and its treatment effect sizes generally comparable to nonsteroidal anti-inflammatory drugs, it appears that this eHealth intervention for HRQOL may be useful for patients with chronic pain during the COVID-19 pandemic.

## Figures and Tables

**Table 1:** Sociodemographic and clinical characteristics of the patients at baseline according to treatment group (*N* = 102) *.

Characteristic	Experimental Treatment (eHealth Intervention)	Control Treatment (Wait List)	*p*
	n = 52	n = 50	
	%	%	
Age (year), mean ± SD	51.3 ± 13.7	50.7 ± 13.0	0.84
Female gender	81	88	0.32
Non-White race	25	18	0.39
Hispanic ethnicity	14	6	0.32
College education or higher	37	32	0.63
Current cigarette smoker	19	8	0.10
Low back pain duration greater than 5 years	71	60	0.24
Ever unemployed or unable to do usual work			
for one month or longer due to low back pain	58	54	0.71
Ever received disability or workers’ compensation			
benefits relating to low back pain	25	28	0.73
Ever involved in a lawsuit or legal claim relating to low back pain	10	10	>0.99
Currently using NSAIDs for low back pain	62	72	0.26
Currently using opioids for low back pain	50	42	0.42
Ever had surgery for low back pain	23	22	0.90
Comorbidities ever reported			
Herniated disc	48	44	0.68
Sciatica	52	58	0.54
Osteoporosis	17	16	0.86
Osteoarthritis	39	48	0.33
Heart disease	10	12	0.70
Hypertension	48	40	0.41
Diabetes mellitus	33	24	0.33
Asthma	23	20	0.71
Depression	64	64	0.95
SPADE cluster score, mean ± SD	61.8 ± 4.2	61.4 ± 4.3	0.67
SPADE scale scores			
Sleep disturbance, mean ± SD	60.8 ± 7.1	61.1 ± 7.7	0.83
Pain interference with activities, mean ± SD	66.4 ± 5.1	66.5 ± 5.7	0.93
Anxiety, mean ± SD	59.6 ± 7.4	58.3 ± 7.7	0.40
Depression, mean ± SD	59.6 ± 7.9	55.9 ± 8.0	0.02
Low energy/fatigue, mean ± SD	62.5 ± 7.9	65.1 ± 6.6	0.07
NRS score, mean ± SD	6.0 ± 1.7	6.2 ± 1.9	0.55
RMDQ score, mean ± SD	17.3 ± 4.2	16.3 ± 5.3	0.28

* Results are displayed as % unless otherwise indicated. NRS denotes numerical rating scale for low back pain intensity; NSAID, nonsteroidal anti-inflammatory drug; RMDQ, Roland-Morris Disability Questionnaire; SPADE, sleep disturbance, pain interference with activities, anxiety, depression, low energy/fatigue.

**Table 2:** Responses to survey on value and utility of the eHealth intervention (*N* = 51) *.

Survey Item	%
Overall value rating of the report (100-point NRS) (mean ± SD)	62.9 ± 29.0
Report was easy to understand after reading the interpretation guide	
Strongly agree	47
Agree	39
Neither agree nor disagree	8
Disagree	4
Strongly disagree	2
Report provided information about quality of life that I did not know	
Strongly agree	24
Agree	55
Neither agree nor disagree	12
Disagree	6
Strongly disagree	4
Patient actions prompted by the report	
Reading or learning more about improving health-related quality of life	77
Beginning a new program to improve health-related quality of life	41

* Results are displayed as % unless otherwise indicated. The survey data were provided by 51 patients in the experimental treatment group who were available at 1 month following randomization. NRS denotes numerical rating scale.

**Table 3:** Changes in primary and secondary outcome measures over 3 months according to treatment group (*N* = 100) *.

Outcome Measure	Experimental Treatment (eHealth Intervention)	Control Treatment (Wait List)	*p*	Effect Size (SMD)
	n = 51	n = 49		
	Mean Change Score (95% CI)	Mean Change Score (95% CI)		
Primary Outcome Measures				
SPADE cluster score	1.2 (0.2–2.2)	0.2 (−1.1–1.5)	0.23	0.24
SPADE scale scores				
Sleep disturbance	1.0 (−0.6–2.6)	0.1 (−1.5–1.8)	0.47	0.15
Pain interference with activities	1.3 (−0.1–2.6)	0.9 (−0.5–2.4)	0.72	0.07
Anxiety	1.2 (−1.0–3.4)	−0.8 (−3.1–1.6)	0.23	0.24
Depression	1.9 (−0.1–3.9)	−1.1 (−3.6–1.4)	0.06	0.37
Low energy/fatigue	0.7 (−1.1–2.5)	1.8 (−0.6–4.2)	0.47	−0.15
Secondary Outcome Measures				
Low back pain intensity (NRS score)	−0.3 (−0.8–0.2)	−0.1 (−0.5–0.3)	0.59	−0.11
Back-related disability (RMDQ score)	0.9 (−0.3–2.1)	−0.4 (−1.2–0.4)	0.07	0.36

* The reported P values are for the differences in mean change scores between groups. The SMDs were computed as the between-group differences in mean change scores/pooled SD of the relevant change score. Positive SMDs favor the experimental treatment. The data were provided by patients who completed the 3-month follow-up encounter. NRS denotes numerical rating scale; RMDQ, Roland-Morris Disability Questionnaire; SMD, standardized mean difference; SPADE, sleep disturbance, pain interference with activities, anxiety, depression, low energy/fatigue.

## Supplementary Materials

The following are available online at https://www.mdpi.com/2227-9032/8/4/381/s1, File S1.
