# Supplementary material for: Feasibility Trial of an eHealth Intervention for Health-Related Quality of Life: Implications for Managing Patients with Chronic Pain During the COVID-19 Pandemic
Source: Healthcare (Basel). 2020 Oct 1;8(4):381. doi: 10.3390/healthcare8040381 (PMC7712291; doi:10.3390/healthcare8040381)
Supplement: Supplementary file 1 [file healthcare-08-00381-s001.pdf]

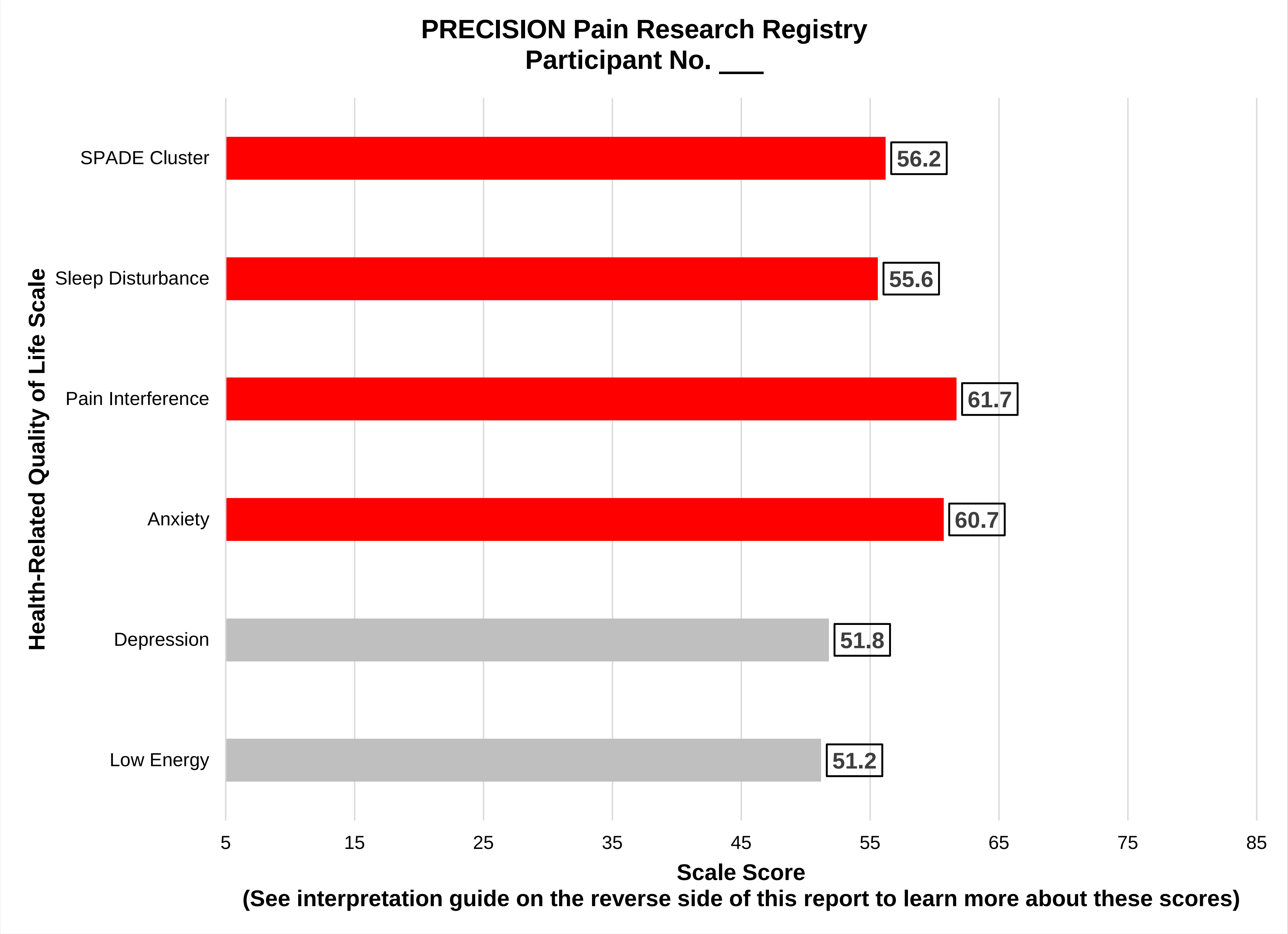
PRECISION Pain Research Registry
Participant No. ___
Health-Related Quality of Life Scale
SPADE Cluster
56.2
Sleep Disturbance
55.6
Pain Interference
61.7
Anxiety
60.7
Depression
51.8
Low Energy
51.2
5
15
25
35
45
55
65
75
85
Scale Score
(See interpretation guide on the reverse side of this report to learn more about these scores)

Interpretation Guide

Background Regarding Health-Related Quality of Life Measures

Thank you for participating in the PRECISION Pain Research Registry and our project on Health-Related Quality of Life. The responses that you provided on your recent survey were analyzed to generate the attached report that summarizes your quality of life. The overall quality of life is measured with the SPADE cluster score, which is composed of the following scales: (1) Sleep Disturbance; (2) Pain Interference with Activities; (3) Anxiety; (4) Depression; and (5) Low Energy. This report may be used along with pain scores and other factors to assess how low back pain affects a person's life. However, it is important to understand that these measures are based only on research questionnaires and they cannot substitute for medical advice that would normally be provided by a licensed physician. You may wish to share the information provided in this report with your physician.

How to Interpret the Scores in this Report

The scores in this report show how a person compares with other adults in the United States population. The scores on the SPADE cluster range from about 20 to 80, with an average of 50. Persons who score high on the SPADE cluster (as indicated by red shading in this report) generally have more overall problems with quality of life than persons with lower scores on the SPADE cluster. Similarly, patients who score high on any of the 5 individual scales generally have more problems with the aspect of health being measured by the scale. For example, a score of 55 or higher (as indicated by red shading rather than gray shading) means that a person reports more sleep disturbance than about 70% of adults in the United States. The higher the score, the greater the level of sleep disturbance. A score of 65 or higher indicates that the person reports more sleep disturbance than about 93% of the adults in the United States. The scores for pain interference, anxiety, depression, and low energy may be interpreted in the same manner as those described for sleep disturbance. The scores on each of the 5 scales that comprise the SPADE cluster may be used to help determine which aspects of health are most in need of improvement.

How You May use this Report

You may use this report to identify aspects of your health that need improvement and then take appropriate action. This may include simple things that you can do on your own ("self-care") to improve your health in any of the 5 reported areas. You may also consider sharing this report with your physician. This may help your physician better understand the relationship between your quality of life and your low back pain and any related problems. In so doing, you may also learn more about other approaches or treatments to improve these aspects of your health.
